# SERS-based rapid susceptibility testing of commonly administered antibiotics on clinically important bacteria species directly from blood culture of bacteremia patients

**DOI:** 10.1007/s11274-023-03717-x

**Published:** 2023-08-17

**Authors:** Yin-Yi Han, Jann-Tay Wang, Wei-Chih Cheng, Ko-Lun Chen, Yi Chi, Lee-Jene Teng, Juen-Kai Wang, Yuh-Lin Wang

**Affiliations:** 1 Department of Anesthesiology, National Taiwan University Hospital, 7 Zhongshan S. Road, Taipei, 100225 Taiwan; 2 Department of Surgery, National Taiwan University Hospital, 7 Zhongshan S. Road, Taipei, 100225 Taiwan; 3 Department of Traumatology, National Taiwan University Hospital, 7 Zhongshan S. Road, Taipei, 100225 Taiwan; 4 Division of Infectious Diseases, Department of Internal Medicine, National Taiwan University Hospital, 7 Zhongshan S. Road, Taipei, 100225 Taiwan; 5 Taiwan National Institute of Infectious Diseases and Vaccinology, National Health Research Institutes, 35 Keyan Road, Zhunan, Miaoli, 35053 Taiwan; 6 Institute of Atomic and Molecular Sciences, Academia Sinica, 1 Roosevelt Road Sec. 4, Taipei, 10617 Taiwan; 7 Department of Clinical Laboratory Sciences and Medical Biotechnology, National Taiwan University, 1, Roosevelt Road Sec. 4, Taipei, 10048 Taiwan; 8 Center for Condensed Matter Sciences, National Taiwan University, 1 Roosevelt Road Sec. 4, Taipei, 106319 Taiwan

**Keywords:** Antimicrobial susceptibility testing, Surface-enhanced Raman scattering, Blood culture, Bacteremia

## Abstract

Bloodstream infections are a growing public health concern due to emerging pathogens and increasing antimicrobial resistance. Rapid antibiotic susceptibility testing (AST) is urgently needed for timely and optimized choice of antibiotics, but current methods require days to obtain results. Here, we present a general AST protocol based on surface-enhanced Raman scattering (SERS-AST) for bacteremia caused by eight clinically relevant Gram-positive and Gram-negative pathogens treated with seven commonly administered antibiotics. Our results show that the SERS-AST protocol achieves a high level of agreement (96% for Gram-positive and 97% for Gram-negative bacteria) with the widely deployed VITEK 2 diagnostic system. The protocol requires only five hours to complete per blood-culture sample, making it a rapid and effective alternative to conventional methods. Our findings provide a solid foundation for the SERS-AST protocol as a promising approach to optimize the choice of antibiotics for specific bacteremia patients. This novel protocol has the potential to improve patient outcomes and reduce the spread of antibiotic resistance.

**Supplementary Information:**

The online version contains supplementary material available at 10.1007/s11274-023-03717-x.

## Introduction

Bloodstream infections (BSI) are a leading infectious syndrome and can lead to sepsis that is a medical emergency with a high mortality rate, resulting in rising costs of its management (Goto and Al-Hasan 2013). In 2019 alone, the estimated global BSI-associated deaths were 2.91 million and 56.2% of the total sepsis-related deaths was caused by bacteremia (G. B. D. Antimicrobial Resistance Collaborators 2022). It is recommended that obtaining blood cultures and lactate levels followed by administration of broad-spectrum antibiotics and 30 ml/kg of crystalloid fluid for hypotension be done within 3 h of presumptive sepsis diagnosis (Evans et al. 2021; Seymour et al. 2017). Timely adequate antibiotic administration after the initial empirical antibiotic administration is crucial for the outcomes of patients with sepsis (Retamar et al. 2012), but conventional bacterium-culture and antimicrobial susceptibility testing (AST) methods take 3–5 days to complete even with the aid of modern automated microbiological analysis systems (Webb et al. 2016). A recently reported retrospective cohort analysis over ten years (2005–2014) showed that 19% of the patients received discordant empirical antibiotic therapy that was associated with increased risk of mortality (adjusted odds ratio 1.46) (Kadri et al. 2021). Empirical antibiotic usage may not be effective and may contribute to the development of antibiotic-resistant bacterial strains. The global deaths in 2019 that were associated with antimicrobial resistance by bacterial pathogens were 4.95 million (Antimicrobial Resistance Collaborators 2022). More specifically, resistant pathogens were isolated in 26.8% of culture-proven sepsis patients included from 2005 to 2014 in a retrospective cohort study (Rhee et al. 2020), while 67% of the patients were administrated with empiric therapy targeted resistant organisms. The study found that both inadequate and unnecessarily broad empiric antibiotics were associated with higher mortality. These studies underline the urgent need for better AST tests to rapidly identify adequate antibiotics for patients with bloodstream infections.

During the past two decades, tremendous progress has been made in the development of rapid microbiological diagnostics, such as matrix-assisted laser desorption ionization-time of flight mass spectroscopy (Oviano and Bou 2019), next-generation sequencing (van Belkum and Dunne 2013), and nucleic acid amplification technologies (Lee et al. 2019). These techniques can provide rapid identification and determine antibiotic susceptibility of causal microorganisms even for those that are non-culturable or of low concentration (Riedel and Carroll 2016). However, the accuracy of these diagnostics may be impaired by unsatisfactory specimen preparation, interference of co-existing human DNA fragments or proteins, or inability to distinguish dead from live bacteria, resulting in high false rates. Furthermore, most genome- or proteome-based methods are not capable of detecting emerging antibiotic-resistant microorganisms and determining the minimum inhibitory concentration (MIC) of antibiotic targeting a specific pathogen. In sum, although these genome- and proteome-based diagnostic methods have provided bacterial speciation readily, their AST performance is not satisfiable in clinical settings.

Surface-enhanced Raman scattering (SERS), being an optical technique, works based on the light field that is resonantly excited by an incident light field in proximity of a metal nanostructure. Upon such resonant excitation, the free electrons inside the metal nanostructure undergo collective motion and build up coherently accumulated field on the surface. The strong local field thus enhances Raman scattering of molecules residing within. Its up-to-date developments and applications have been reviewed (Langer et al. 2020). It has also been demonstrated for rapid microbiological testing with minimal sample preparation (Wang et al. 2018) and has been used for classification of bacterial pathogens and for investigation of bacterial antibiotic resistogram (Hassanain et al. 2022). While identification of bacteria by SERS has been demonstrated (Galvan and Yu 2018; Jarvis et al. 2006; Jarvis and Goodacre 2004), its clinical application remains challenging due to problems such as low reproducibility of SERS signal. The low reproducibility of SERS has been investigated by many groups, as reviewed by Jarvis et al. (Jarvis et al. 2008). Its main cause is the non-repeatable and unstable hot junctions (induced by plasmonic coupling between two adjacent metal nanostructure within 10 nm) in the sporadic segregates of metal colloids that have been commonly used in previous SERS studies, thus rendering the results of SERS mostly dubious and prohibiting its deployment in actual applications. The measured SERS signal is often varied by tens and even more than a hundred percentages. Additionally, low concentration of target bacteria and interfering substances in clinical samples further complicates the process (Ranjith et al. 2014). Based on hexagonally packed nanochannels fabricated with anodization on aluminum, we have developed a SERS substrate made of two-dimensional ordered Ag nanoparticles grown by electrodeposition in these nanochannels (dubbed AgNP/AAO) (Wang et al. 2006). Upon resonant excitation, the electromagnetic field enhanced at the hot junction between adjacent Ag nanoparticles can effectively amplify the SERS signal of analytes situated at the substrate. In comparison with other SERS substrates, since Ag nanoparticles are firmly fixed in the ordered hexagonally packed nanochannels in anodic aluminum oxide, the hot junctions are uniformly and stably distributed in the substrate. As a consequence, the SERS signal acquired with use of the AgNP/AAO substrate is varied within 10% over a SERS-active region of 6 cm × 2 cm and is stable for at least one hour (Dvoynenko et al. 2021), allowing for quantitative SERS measurements. Based on this substrate, we have developed a SERS-based platform to identify *Mycobacterium* species (Cheng et al. 2021), to determine antibiotic susceptibility of two bacterium species (Han et al. 2020; Liu et al. 2016), monitor environmental pollution (Dvoynenko et al. 2021), and to identify food adulteration (Lian et al. 2015).

The SERS spectra of bacteria determined in our studies (Han et al. 2020; Liu et al. 2009, 2016) are similar to those obtained by other investigators (Boardman et al. 2016; Premasiri et al. 2005; Zhao et al. 2018), indicating their common origins. Subsequent in-depth studies showed that bacterial SERS signals stem from secreted purines and their derivatives (e.g., adenine, hypoxanthine, xanthine, guanine, uric acid, and adenosine monophosphate) (Chiu et al. 2018; Premasiri et al. 2016). Premasiri et al. (Premasiri et al. 2016), for example, showed that the SERS signal of *S. aureus* is mainly contributed by adenine, that of *E. faecalis* is dominated by hypoxanthine and that of *A. baumannii* mainly reflects xanthine. They also fit the acquired SERS spectra of bacteria with the combination of the SERS spectra of several purines and derivatives and found, for example, that the SERS spectrum of *E. coli* is composed of hypoxanthine (49%), xanthine (25%), adenine (14%), and guanine (12%). Moreover, Chiu et al. (Chiu et al. 2018) studied the dynamical release of these molecules in water by *S. aureus* and *E. coli* with liquid chromatography-mass spectrometer and found that their release has reached saturation after 1 h. These studies also showed that the SERS spectra of Gram-positive bacteria exhibit a common prominent peak at 730 cm^−1^ while the corresponding prominent peak for Gram-negative bacteria resides at 724 or 654 cm^−1^ (Liu et al. 2016). The 730-cm^−1^ peak is contributed by the secreted adenine from Gram-positive bacteria, while the 724 (654)-cm^−1^ peak is attributed to hypoxanthine (xanthine) secreted by Gram-negative bacteria (Chiu et al. 2018; Premasiri et al. 2016). Based on this finding, we demonstrated that these SERS peaks can act as biomarkers to reflect the amount of the respective live bacteria after antibiotic treatment and thus developed a SERS-based AST method (referred as SERS-AST) (Liu et al. 2016). With this method, we have performed a proof-of-principle study (Han et al. 2020) on *Staphylococcus aureus* and *Escherichia coli* isolates from blood cultures to determine their susceptibility to oxacillin (OXA) and cefotaxime (CTX), respectively. The consistency of our SERS-AST results of *S. aureus*-OXA and *E. coli*-CTX combinations was 93% compared with those of conventional AST methods. This success was made possible with the development of a sample preparation procedure—lysis with ACK buffer and sonication followed by repetitive dilution plus centrifugation—that effectively remove the interference from other concurrently present constituents in blood-culture samples. Can the SERS-AST method, acting on the basis of the prompt variation in bacterial metabolism in response to antibiotic treatment (Belenky et al. 2015; Zampieri et al. 2017), be applicable to other bacterium-antibiotic combinations, considering that their metabolic responses are generally dissimilar owing to diverse bacterial metabolisms and distinct mechanisms of drug action? This study was aimed to answer this question. The answer would help develop a general SERS-AST method that can be implemented into the diagnostic flow of the clinically relevant bacterial species to determine their resistant to various antibiotics.

Two criteria were used to select bacteria for this demonstrative study. First, according to the SENTRY Antimicrobial Surveillance Program (Diekema et al. 2019), the ten most common bacterial pathogens from 1997 to 2016 were *Staphylococcus aureus* (20.7%), *Escherichia coli* (20.5%), *Klebsiella pneumoniae* (7.7%), *Pseudomonas aeruginosa* (5.3%), *Enterococcus faecalis* (5.2%), *Staphylococcus epidermidis* (3.8%), *Enterobacter cloacae* (2.9%), *Streptococcus pneumoniae* (2.8%), *Enterococcus faecium* (2.8%), and *Acinetobacter baumannii* (2.0%). Second, in 2017, WHO published its first ever list of antibiotic-resistant “priority pathogens” of 12 families of bacteria. According to this list, *A. baumannii*, *P. aeruginosa*, and *Enterobacteriaceae* are of most critical priority, followed by *E. faecium*, *S. aureus*, *Helicobacter pylori*, *Campylobacter* spp., *Salmonellae*, and *Neisseria gonorrhoea*e (World Health Organization 27 February 2017). Based on these two criteria, we chose four Gram-positive species (*S. aureus*, *S. epidermidis*, *E. faecalis* and *E. faecium*) and four Gram-negative species (*E. coli*, *E. cloacae*, *K. pneumoniae* and *A. baumannii*) to be the targets of this study. Seven frequently administrated antibiotics (Mermel et al. 2001) with different mechanisms of action were applied to the blood-culture isolates of these bacterial species according to their respective recommendations.

This report first presents methods utilized in the study, including study design, preparation of blood-culture samples, treatment of antibiotics, SERS assay and spectral analysis, and receiver operating characteristic (ROC) analysis. The SERS biomarker signals of the blood-culture samples of totally 20 bacterium-antibiotic combinations at different antibiotic concentrations illustrate the distinctive responses of the susceptible and resistant strains of each bacterium species after the antibiotic treatment of two or three hours. The efficacy of the SERS-AST protocol is demonstrated by its rates of agreement with those obtained from VITEK 2 (a widely deployed AST diagnostic system) for each bacterium-antibiotic combination. Four issues are then discussed. First, the slower antibiotic mode of action causes the lower agreement rates of the samples treated with of levofloxacin (a quinolone antibiotic). Second, the optical density concurrently measured after the antibiotic treatment shows inconsistent phenotypical response of bacteria. Third, the relatively poor agreement of *K. pneumoniae* is associated with the reduced permeability of its cell envelope. Fourth, future improvements of this study are presented.

## Materials and methods

### Study design

The goal of this study is to obtain the AST outcomes of different bacterium-antibiotic combinations with the SERS-AST protocol and to reveal the possible complications involved owing to the potentially differing metabolic responses upon antibiotic treatment. The four chosen Gram-positive bacteria (*S. aureus*, *S. epidermidis*, *E. faecalis* and *E. faecium*) were treated with four antibiotics—oxacillin (OXA), levofloxacin (LVX), vancomycin (VAN) and ampicillin (AMP), while the four chosen Gram-negative bacteria (*E. coli*, *E. cloacae*, *K. pneumoniae* and *A. baumannii*) were treated with four antibiotics—cefotaxime (CTX), ceftazidime (CAZ), levofloxacin (LVX) and imipenem (IPM). The eight bacterial species are most frequently isolated bacterial species from the blood culture of patients (Diekema et al. 2019; Infection Control Center 2015), while the seven drugs are commonly used for treatment of bloodstream infections (Mermel et al. 2001). VAN is a glycopeptide antibiotic, while OXA, AMP, CTX, CAZ and IPM are *ꞵ*-lactam antibiotics. LVX, as a quinolone antibiotic, is effective in both Gram-positive and Gram-negative species and was applied to all the bacterial species. The SERS-AST results were verified with those obtained by an automated microbial diagnostic system (VITEK 2, bioMérieux) that is widely employed in many clinical microbiology laboratories around the world owing to its high AST accuracy.

### SERS-AST

**Fig. 1:**
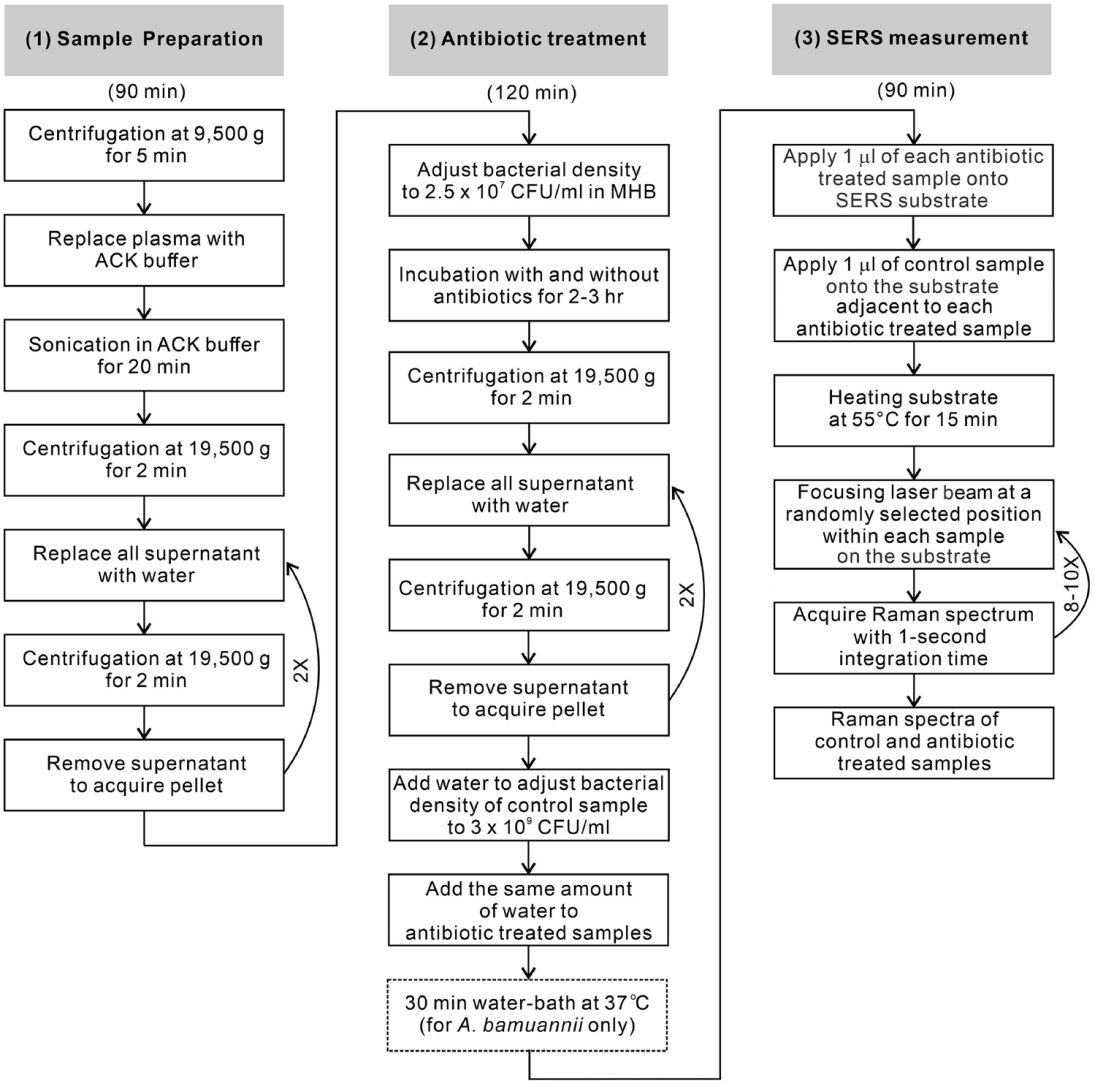
Workflow of SERS-AST comprises three parts: (1) sample preparation, (2) antibiotic treatment and (3) SERS measurement and their separate turnaround times

The laboratory workflow of SERS-AST is illustrated in Fig. 1 and is composed of three parts: sample preparation, antibiotic treatment and SERS measurement. Their turnround times are 90, 120 and 90 min, totaling 5 h. They are followed by spectral signal analysis and receiver operating characteristic analysis. These steps are detailed in the following.

### Sample preparation

For each SERS-AST, 5mL of blood culture broth that grew a single species of bacteria was obtained after bacterial identification was done using matrix-assisted laser desorption ionization-time of flight mass spectroscopy (MALDI Biotyper, Bruker), and the AST might have been performed or completed using VITEK 2. Since hemoglobin dominates the SERS signal of blood-culture samples (Xu et al. 1999), red blood cells were selectively lysed with ammonium-chloride-potassium (ACK) buffer (Phillips et al. 1983) followed by ultrasonication to facilitate sonoporation (Lentacker et al. 2014), resulting in disruption of cell membranes and release of cellular contents. The sonicated mixture then underwent a wash procedure (centrifugation at 19,500 g for 2 min followed by replacement of supernatant with deionized water) for three times to remove other constituents in blood components, leaving viable bacteria pellet for subsequent antibiotic treatment. Our previous study (Han et al. 2020) showed that the residual SERS signal of hemoglobin would be negligible after this pretreatment procedure.

### Antibiotic treatment

The bacterial pellet was diluted with Mueller Hinton broth (MHB) to 2.5 × 10^7^ CFU/ml. The lyophilized antibiotics were dissolved separately to yield four serial drug concentrations (${D}_{1}$–${D}_{4}$), where ${D}_{i}=2\times {D}_{i-1}$, according to the international performance standards of AST set by CLSI (CLSI 2020) (Table S1). They were chosen to cover the concentrations for determining resistogram—i.e., the drug concentrations corresponding to susceptible (${D}_{\text{S}}$), intermediate concentration (${D}_{\text{I}}$) and resistant (${D}_{\text{R}}$). For each test, five cell culture tubes were filled individually with the bacterial suspension. Four of the tubes were then loaded with antibiotic solutions of the four serial concentrations, leaving one filled with deionized water as control. Finally, all the tubes were incubated in the thermostat at 37 °C for 2 h so that the optical density value at 600 nm (OD_600_) of the control sample was checked at the end of incubation with the target larger than 1 to ensure enough bacterial amount. The incubation time might be prolonged to 3 h if the growth rate of some bacteria is comparatively lower, such as some samples of *E. faecium*, *E. faecalis*, *S. epidermidis* and *A. baumannii*. The antibiotic-cultured samples underwent centrifugation followed by the wash procedure three times to remove the drug and the culture medium. The concentrations of all the bacterial samples were adjusted to 3 × 10^9^ CFU/ml for SERS measurements. An additional step of 30-minute water bath at 37℃ was adopted specifically for *A. baumannii* to boost its SERS signal.

### SERS assay and spectral analysis

The substrates for SERS were silver nanoparticles embedded in nanochannels of anodic aluminum oxide (AgNP/AAO) and were fabricated by anodization-etching-electroplating on glass slides (Wang et al. 2006). For the SERS assay, an AgNP/AAO slide, bearing a SERS-active area of 5.5 cm × 2.5 cm, was placed inside an aluminum trough. A 1-mm thick aluminum plate with 3 rows of 8 holes of 1.5-mm in diameter separated from each other by 2.5 mm was hung on the edges of the trough covering the AgNP/AAO slide 7 mm above. A calibrated pipet then injected one microliter of a bacterial suspension (3 × 10^9^ CFU/ml) onto the slide with its pipet tip being protruded through and fixed on the hole, resulting in a circular droplet on the AgNP/AAO slide. In this way, the constant separation between the pipet tip apex and the slide surface ensured a constant diameter (~ 1.5 mm) for each sample droplet. The droplets on the central SERS-active region of the AgNP/AAO slide were arranged so that there was always a control-sample droplet adjacent to any sample droplet (Figure S1). The SERS signal obtained on any sample droplet could be compared with that obtained on an adjacent control-sample droplet. The slide with the sample droplets was then removed from the trough and placed on a hot plate to dry the droplets at 55 °C for 15 min before SERS measurements. As a result, the bacteria in the sample droplet lost their viability (confirmed with agar-plate culture) and the secreted molecules from the bacteria were firmly attached on the surface of the AgNP/AAO slide. As shown in Figure S1, there is still large unused area of the SERS-active region, allowing for more sample droplets treated with other antibiotics.

The Raman instrument for the SERS measurements was composed of a He–Ne laser emitting at 632.8 nm, an upright optical microscope, a Raman probe (Superhead, Horiba), a spectrometer, and a thermoelectric-cooled charge-coupled camera. The typical laser irradiation power density at the substrate surface was about 1 × 10^5^ mW/cm^2^, while the Raman spectra were acquired with an integration time of 1 s. The backscattered light from the sample on the AgNP/AAO slide was collected by the same objective lens, spectrally filtered by a long-pass filter in the Raman probe, and then transmitted via another optical fiber to the spectrometer and the CCD for spectral recording and analysis. The spectral calibration using a Ne lamp resulted in a resolution and error were 20 and 3 cm^−1^, respectively. To mitigate the variation in individual SERS spectra caused by the possible coffee-ring effect in dried sample droplets, multiple Raman measurements were performed at 8–10 randomly selected laser-focused positions within each sample spot on the SERS substrate for signal averaging (Zang et al. 2019) in each dried sample droplets. Before signal averaging, similar to our previous works (Han et al. 2020; Liu et al. 2016), the outlier spectra that exhibited dissimilar spectral patterns and excessively large backgrounds were removed and the left-over spectra underwent baseline removal.

The prominent peaks of Gram-positive and Gram-negative bacteria at 730 and 724 cm^−1^, respectively, were considered as their corresponding biomarkers. Their signal variations were used to determine the respective bacterial responses to antibiotic treatment. Different from the commonly secreted adenine and hypoxanthine by Gram-positive and Gram-negative bacteria, respectively, the dominant secretion of *A. baumannii* is xanthine (Premasiri et al. 2016), conferring a prominent peak at 654 cm^−1^. As a consequence, this peak was used as the biomarker of *A. baumannii*. The signal ratio of the biomarker of tested bacteria was calculated by dividing the peak signal of the antibiotic-treated sample droplet by that of the adjacent non-treated control-sample droplet: ${r}_{{\nu }_{\text{b}\text{m}}}={S}_{{\nu }_{\text{b}\text{m}}}/{S}_{{\nu }_{\text{b}\text{m}}}^{0}$, where ${S}_{{\nu }_{\text{b}\text{m}}}$ and ${S}_{{\nu }_{\text{b}\text{m}}}^{0}$ are the signal strengths of the biomarker peak at ${\nu }_{\text{b}\text{m}}$ with and without the antibiotic treatment, respectively. For each bacterial-antibiotic combination, four SERS-AST signal ratios corresponding to four different antibiotic concentrations were obtained. The SERS uniformity test with adenine (10^−4^ M) of the AgNP/AAO slide showed that the average standard deviation across the SERS-active area was 15% (Han et al. 2020; Liu et al. 2016). The difference between the SERS enhancement factors between the two 2.5 mm-separated droplet sites on the SERS-active area of the AgNP/AAO substrate would be even smaller. In fact, the signal standard variation of the SERS biomarker signals measured at the four control sample droplets was typically less than 10%. The high repeatability of our bacterial SERS signal is crucial for the ROC analysis of the obtained signal ratios obtained from the bacterial samples treated with different antibiotic concentrations.

### Receiver operating characteristic analysis

The flow chart of the ROC analysis of the SERS-AST results used in this study (Figure S2) generalizes the one reported in our previous work (Han et al. 2020) and is delineated below. For a specific bacterium-antibiotic combination, the highest drug concentration just below its resistant drug concentration, ${D}_{\text{R}}$, or its intermediate drug concentration if it exists, ${D}_{\text{I}}$, according to the CLSI standard is designated as the break-point concentration for susceptibility discrimination—i.e., ${D}_{\text{B}\text{P}}={D}_{\text{R}}/2$ or ${D}_{\text{I}}/2$ (Table S1). If the signal ratios of the antibiotic-treated samples with the drug concentrations, which are higher than or equal to ${D}_{\text{B}\text{P}}$, are all smaller than a chosen cut-off signal ratio ${r}^{*}$—i.e.,

1 $${r}_{{D}_{\text{B}\text{P}}},\cdots ,{r}_{{D}_{4}}\le {r}^{*}$$

the bacterial sample is considered to be susceptible to the drug. On the other hand, if Eq. (1) is invalid, the bacterial sample is considered to be resistant to the drug. The AST result is then compared with the result from VITEK 2 which is routinely used in many clinical laboratories for antibiotic susceptibility testing and its effectiveness has been repeatedly compared to other reference methods such as broth microdilution (Klare et al. 2019; Peterson et al. 2011; Pfaller et al. 2007), agar dilution (Lee et al. 2013), double-disk diffusion (Filippin et al. 2014), and PCR for mecA (Torres et al. 2008). The comparison then gives TRUE POSITIVE, FALSE POSITIVE, TRUE NEGATIVE or FALSE NEGATIVE. The whole procedure then runs through all the samples of the specific bacterium-antibiotic combination, yielding the numbers of TRUE POSITIVE, FALSE POSITIVE, TRUE NEGATIVE and FALSE NEGATIVE (${n}_{\text{T}\text{P}}$, ${n}_{\text{F}\text{P}}$, ${n}_{\text{T}\text{N}}$ and ${n}_{\text{F}\text{N}}$, respectively) from the whole *n* samples ($n={n}_{\text{T}\text{P}}+{n}_{\text{F}\text{P}}+{n}_{\text{T}\text{N}}+{n}_{\text{F}\text{N}}$) and thus true positive rate—Sensitivity, ${R}_{\text{T}\text{P}}={n}_{\text{T}\text{P}}/\left({n}_{\text{T}\text{P}}+{n}_{\text{F}\text{N}}\right)$—and true negative rate—Specificity, ${R}_{\text{T}\text{N}}={n}_{\text{T}\text{N}}/\left({n}_{\text{T}\text{N}}+{n}_{\text{F}\text{P}}\right)$. For each ${r}^{*}$ varied from 0 to the highest measured signal ratio obtained from the bacterial samples, a ROC curve is thus obtained. The calculated area under the curve (AUC) is considered as an effective assessment of the accuracy. The optimal cut-off signal ratio, ${r}_{\text{O}\text{P}}^{*}$, for each bacterium-antibiotic combination is the one that maximizes the Youden’s indices—$J=\text{S}\text{e}\text{n}\text{s}\text{i}\text{t}\text{i}\text{v}\text{i}\text{t}\text{y}+\left(\text{S}\text{p}\text{e}\text{c}\text{i}\text{f}\text{i}\text{c}\text{i}\text{t}\text{y}-1\right)$—and ensures that all the resistant samples were validified to avoid any very major error (i.e., the bacterial sample resistant to the antibiotic is regarded to be susceptible to it). If there is a range of ${r}^{*}$ values that meet the above two criteria, its middle value is chosen for ${r}_{\text{O}\text{P}}^{*}$. The comparison between the results of SERS-AST and that of VITEK 2 of each bacterium-antibiotic combination results in its agreement rate.

From March 2016 to June 2019, a total of 164 bacterial isolates from blood samples were analyzed, including *S. aureus* (*n* = 20), *S. epidermidis* (*n* = 21), *E. faecalis* (*n* = 20), *E. faecium* (*n* = 21), *E. coli* (*n* = 20), *E. cloacae* (*n* = 20), *K. pneumoniae* (*n* = 21), and *A. baumannii* (*n* = 21). Three samples failed to generate analyzable SERS signals, including one *S. epidermidis* and one *E. faecium* sample with insufficient growth and one *A. baumannii* sample that generated unrecognizable SERS signals. Results of three *K. pneumoniae*-IPM tests were also excluded because of improper antibiotic preparation. There were six bacterium-antibiotic combinations (including *S. aureus*-VAN, *S. epidermidis*-VAN, *E. faecalis*-AMP, *E. faecalis-*VAN, *E. coli*-IPM, *E. cloacae*-LVX, and *E. cloacae*-IPM) excluded from the ROC analysis due to insufficient numbers (< 3) of antibiotic-resistant samples. A total of 401 SERS spectra underwent the ROC analysis.

## Results

The SERS-AST results of blood-culture Gram-positive isolates are presented first, followed by those of blood-culture Gram-negative isolates. The signal-ratio distributions of their respective biomarkers under different designated antibiotics at their corresponding four drug concentrations show the distinctive antibiotic responses of the susceptible and resistant species. The SERS-AST results obtained from the ROC analysis based on the collected blood-culture isolates are summarized in the corresponding tables. Besides, boosting the SERS signal of *A. baumannii* by heating in water is demonstrated. In the end, the dependence of the SERS signal of susceptible bacterial isolates on the treatment time of LVX is presented to illustrate how its slower mechanism of action affects the AST outcome.

### SERS-AST of gram-positive bacteria

**Fig. 2:**
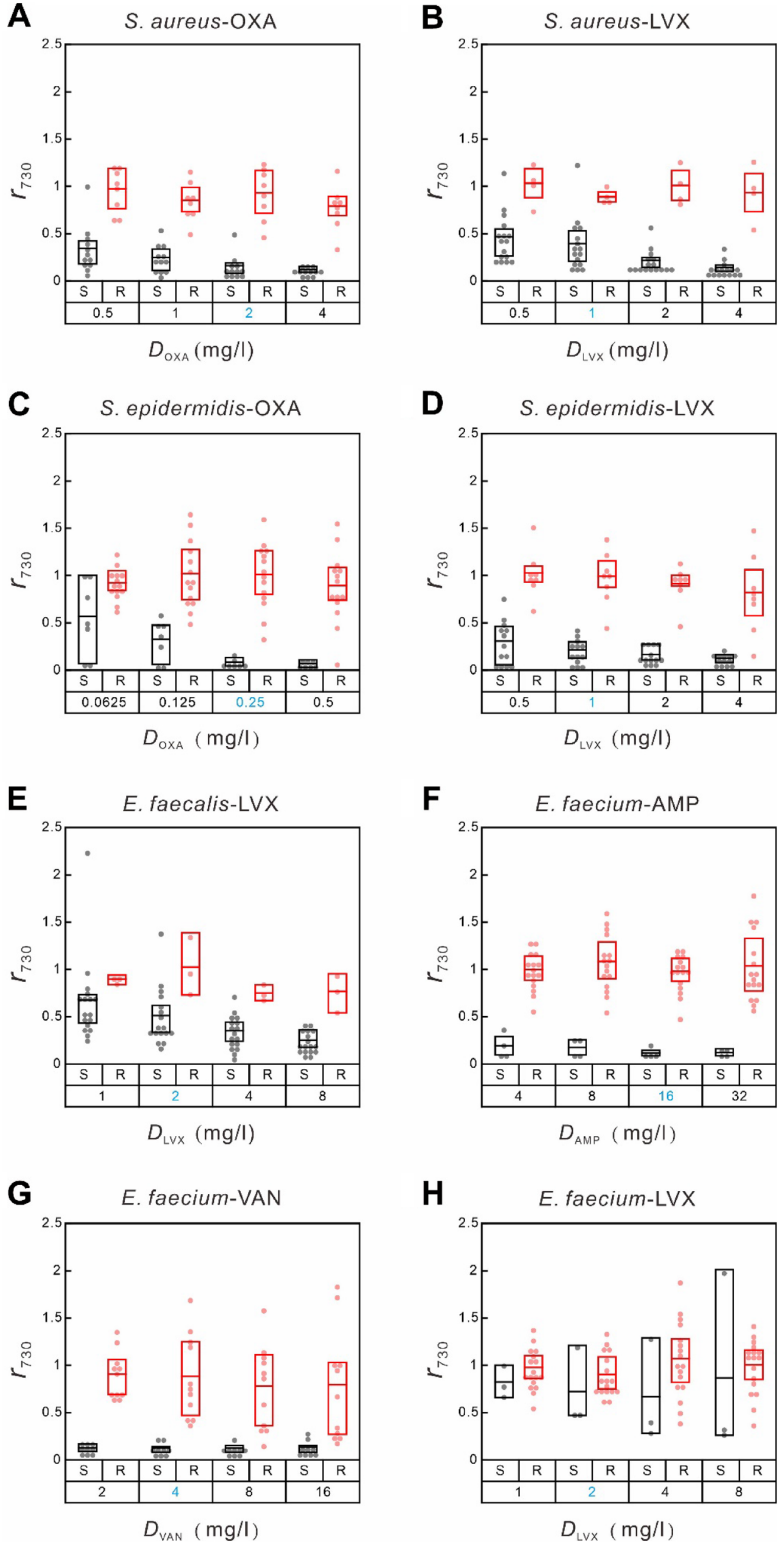
Box-dot plots of SERS biomarker signals of Gram-positive bacteria treated with various concentrations of antibiotics. **A ***S. aureus* with oxacillin (OXA); **B ***S. aureus* with levofloxacin (LVX); **C ***S. epidermidis* with OXA; **D ***S. epidermidis* with LVX; **E ***E. faecalis* with LVX; **F ***E. faecium* with ampicillin (AMP); **G ***E. faecium* with vancomycin (VAN); **H ***E. faecium* with LVX. The Y-axis shows SERS-AST signal ratios (${\varvec{r}}_{730}$) obtained by dividing the signal strength at 730 cm^-1^ of the antibiotic-treated sample by that of the non-treated control sample. The X-axis represents antibiotic concentrations, with blue numbers indicating the break-point concentrations of the corresponding antibiotics (Table S1). The red and black boxes represent the 25th to 75th percentiles of the resistant and susceptible blood-culture isolates, respectively, with mean values indicated by horizontal lines. Each dot symbolizes the data of an individual sample

As the SERS spectra of the chosen Gram-positive bacteria (shown in Figure S3) consistently exhibit a prominent peak at 730 cm^−1^, the ratio of its signal strength of the antibiotic-treated sample to that of the untreated control sample (${r}_{730}$) was used to analyze the antibiotic response of each Gram-positive bacterium. Given our previous study of the *S. aureus*-OXA combination (Han et al. 2020; Liu et al. 2016), the experiment was repeated with two additional antibiotics (LVX and VAN). Table 1 shows the accumulated susceptible and resistant blood-culture isolates of the three antibiotics. Since VAN-resistant sample was not available during this study, the ROC analysis under the VAN treatment was not performed. The distributions of ${r}_{730}$ of the susceptible and resistant samples under the treatment of OXA and LVX at their respective concentrations show similar behavior (Fig. 2): ${r}_{730}$ decreases to small values for the susceptible samples as the drug concentration is above certain value, while it remains at high values for the resistant samples. This result indicates that the response of the bacterial SERS signal of *S. aureus* treated with LVX is similar to that treated with OXA. The ROC analysis was performed on both the OXA and LVX-treated samples (ROC curves shown in Figure S4**A**). As shown in Table 1, the AUC values in the two cases are near unity (0.99 and 0.95 for OXA and LVX, respectively), reflecting their good classification performance, and their agreement rates between the results of SERS-AST and VITEK 2 are both 95%. There is one major-error sample in each of these two bacterium-antibiotic combinations. However, the optimal cut-off signal ratio (${r}_{\text{O}\text{P}}^{*}$) extracted from the LVX-treated samples is significantly higher than that from the OXA-treated samples (0.83 vs. 0.36). This difference can be understood from the signal-ratio distributions of the *S. aureus*-OXA and *S. aureus*-LVX combinations (Fig. 2A and **B**) at their respective break-point drug concentrations (${D}_{\text{B}\text{P}}$ = 2 and 1 mg/l, respectively), because the ${r}_{730}$ value at ${D}_{\text{B}\text{P}}$ decides whether the blood-culture isolate is susceptible or resistant to the antibiotic according to Eq. (1). Note that the ${r}_{730}$ values of the susceptible *S. aureus* isolates treated with OXA of 2 mg/l are well below 0.25 while those of the resistant isolates are mostly above 0.75; the ${r}_{730}$ values of the susceptible *S. aureus* isolates treated with LVX of 1 mg/l are mostly around 0.5 while those of the resistant isolates are mainly above 0.9. Accordingly, the obtained ${r}_{\text{O}\text{P}}^{*}$ for the *S. aureus*-OXA combination (0.36) is expectantly about 0.5 while that for the *S. aureus*-LVX combination (0.83) is higher than 0.7. That is, the change of ${r}_{730}$ of the susceptible *S. aureus* isolates under the treatment of LVX is comparatively smaller than that under the treatment of OXA for a reason discussed later.

**Table 1:** SERS-AST results of gram-positive bacteria. ***N***_**S**_ and ***N***_**R**_ stand for numbers of susceptible and resistant cases, respectively

Bacterium	Antibiotic	${N}_{\text{S}}$		${N}_{\text{R}}$	${r}_{\text{O}\text{P}}^{*}$	AUC	${N}_{\text{D}\text{A}}$	${R}_{\text{A}}$ (%)
*S. aureus*	OXA	12	8		0.36	0.99	1	95
	LVX	16	4		0.83	0.95	1	95
* S. epidermidis*	OXA	6	14		0.25	1	0	100
	LVX	12	8		0.43	0.99	1	95
*E. faecalis*	LVX	17	3		0.68	0.90	3	85
*E. faecium*	LVX	3	17		0.61	0.67	1	95
	VAN	4	16		0.33	1	0	100
	AMP	9	11		0.44	1	0	100

${\varvec{r}}_{\mathbf{O}\mathbf{P}}^{\varvec{*}}$ is the optimal cut-off SERS-AST signal ratio. ${\varvec{r}}_{\mathbf{O}\mathbf{P}}^{\varvec{*}}$ is the optimal cut-off signal ratio obtained from ROC analysis. AUC is the area under the ROC curve. ${\varvec{N}}_{\mathbf{D}\mathbf{A}}$ is the number of isolates that show a SERS-AST result disagreeing with its VITEK 2 result. ${\varvec{R}}_{\mathbf{A}}$ is the agreement rate and is equal to ${\varvec{N}}_{\mathbf{D}\mathbf{A}}/\left({\varvec{N}}_{\mathbf{S}}+{\varvec{N}}_{\mathbf{R}}\right)$

Similar to the *S. aureus* samples, there was no VAN-resistant *S. epidermidis* sample. For *E. faecalis*, OXA- and VAN-resistant sample were both absent, too. On the other hand, AMP-, VAN- and LVX-resistant *E. faecium* samples were obtained. The corresponding distributions of ${r}_{730}$ of the susceptible and resistant samples under the treatment of the antibiotics at their respective concentrations also exhibit a common behavior (Fig. 2) akin to the combinations with *S. aureus*, except the *E. faecium*-LVX combination. The ROC analysis was thus performed on all these combinations. All the analysis results (${r}_{\text{O}\text{P}}^{*}$, AUC, ${N}_{\text{D}\text{A}}$ and ${R}_{\text{A}}$) obtained from the corresponding ROC curves (Figure S4) are shown in Table 1. All the agreement rates are higher than or equal to 85%, indicating that the SERS-AST protocol was also successfully demonstrated on the other three Gram-positive bacterial species treated with the corresponding antibiotics. There is only one major-error sample in each of *S. epidermidis*-LVX and *E. faecium*-LVX combinations, resulting in 95% agreement in both cases. On the other hand, there are three major-error samples in the *E. faecalis*-LVX combination, resulting in 85% agreement. Note that similar to the signal-ratio distributions of the *S. aureus*-LVX combination, the ${r}_{730}$ values of the susceptible at the corresponding break-point drug concentration (2 mg/l) reside around 0.5 (Fig. 2E). According to the SERS-AST results of the four Gram-positive bacterium species, their agreement rates obtained with the treatment of LVX are relatively lower than those with other antibiotics (except the *E. faecium*-LVX combination) and the lower agreement rate is correlated with the smaller decrease in ${r}_{730}$ of their isolates susceptible to the treatment of LVX at the break-point drug concentration. The examination of the inferior performance of the LVX-treated cases will be presented later. The exception to the statement above is the SERS-AST result of the *E. faecium*-LVX combination: its AUC is 0.67 and its ${R}_{\text{A}}$ is 95%. Such anomalous behavior—despite the low AUC value in the *E. faecium*-LVX combination, its ${R}_{\text{A}}$ value is still quite high—can be understood from the spreading ${r}_{730}$ distributions of the *E. faecium* isolates susceptible and resistant to LVX (Fig. 2H). With only three resistant isolates (Table 1), it is relatively easy to locate a ${r}_{\text{O}\text{P}}^{*}$ from the obtained ROC curve (Figure S4**D**) to achieve a high ${R}_{\text{A}}$ value. This large distribution of the ${r}_{730}$ distribution of the *E. faecium*-LVX combination will be further discussed later.

### SERS-AST of gram-negative bacteria

**Fig. 3:**
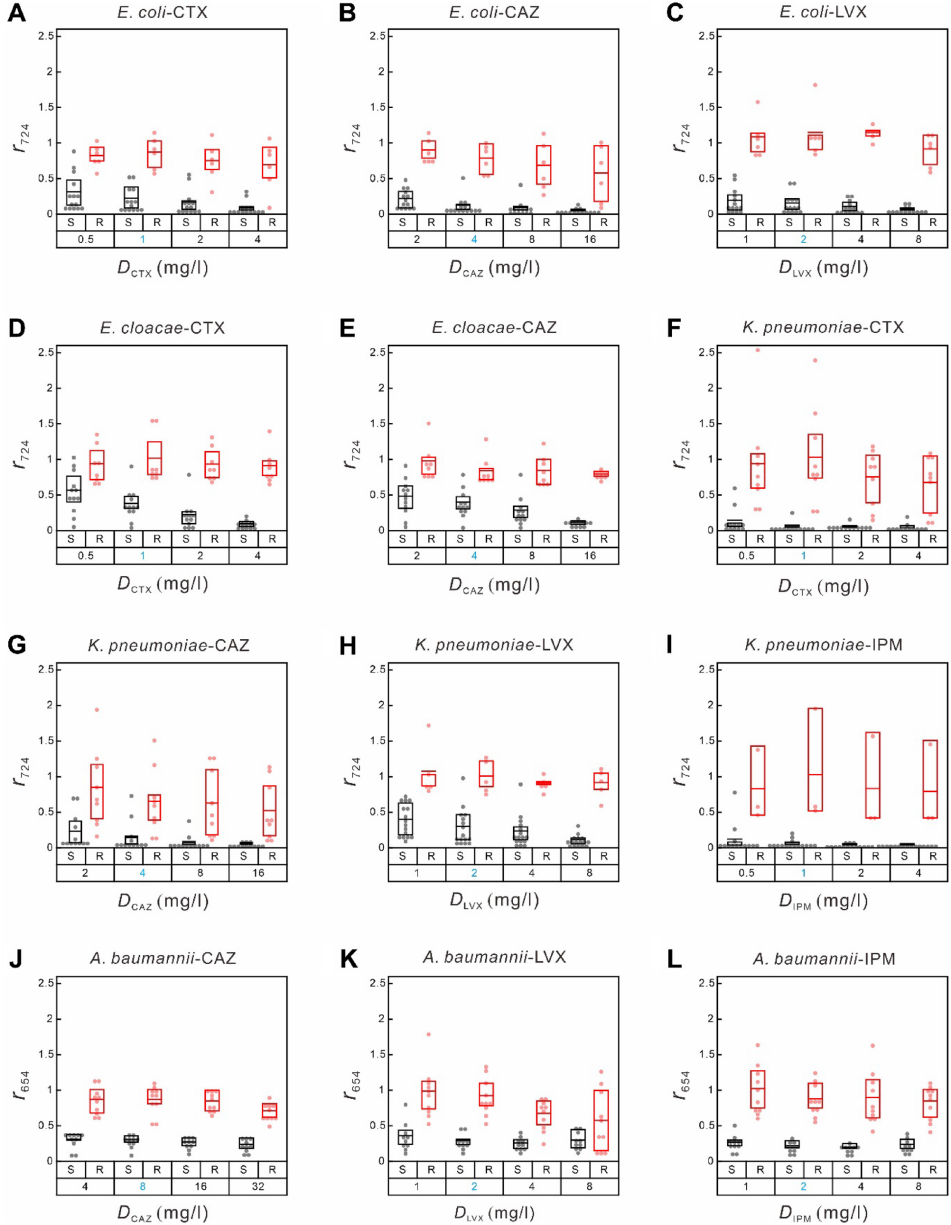
Box-dot plots of SERS biomarker signals of Gram-negative bacteria treated with various concentrations of antibiotics. **A ***E. coli* with cefotaxime (CTX), **B ***E. coli* with ceftazidime (CAZ), **C ***E. coli* with levofloxacin (LVX), **D ***E.* cloacae with CTX, **E ***E. cloacae* with CAZ, **F.***K. pneumoniae* with CTX, **G. ***K. pneumoniae* with CAZ, **H ***K. pneumoniae* with LVX, **I ***K. pneumoniae* with imipenem (IPM), **J ***A. baumannii* with CAZ, **K ***A. baumannii* with LVX, **L ***A. baumannii* with IPM. For **A–I**, the Y-axis shows SERS-AST signal ratios (${\varvec{r}}_{724}$) obtained by dividing the signal strength at 724 cm^-1^ of the antibiotic-treated sample by that of the non-treated control sample. For **J–L**, the Y-axis shows SERS-AST signal ratios (${\varvec{r}}_{654}$) obtained by dividing the signal strength at 654 cm^-1^ of the antibiotic-treated sample by that of the non-treated control sample. The X-axis represents antibiotic concentrations, with blue numbers indicating the break-point concentrations of the corresponding antibiotics (Table S1). The red and black boxes represent the 25th to 75th percentiles of the resistant and susceptible blood-culture isolates, respectively, with mean values indicated by horizontal lines. Each dot symbolizes the data of an individual sample

According to the obtained SERS spectra of the chosen Gram-negative bacteria (shown in Figure S5), the prominent peak of *E. coli*, *E. cloacae* and *K. pneumoniae* is at 724 cm^−1^ while that of *A. baumannii* is at 654 cm^−1^. As a consequence, the ratio of the signal strength of the biomarker at 724 cm^−1^ (${r}_{724}$) of the antibiotic-treated sample of *E. coli*, *E. cloacae* or *K. pneumoniae* to that of the untreated control sample (${r}_{724}$) was used to analyze its antibiotic response, while that at 654 cm^− 1^ (${r}_{654}$) was adopted for *A. baumannii*. Our previous study (Han et al. 2020; Liu et al. 2016) showed the effectiveness of this signal ratio of blood-culture *E. coli* isolates as an indicator to determine the antibiotic susceptibility and MIC of CTX. The SERS-AST experiment was similarly repeated in this work with three additional antibiotics (CAZ, LVX and IPM). Since there was no IPM-resistant *E. coli* isolate collected, no ROC analysis was performed in the *E. coli*-IPM combination. The distributions of ${r}_{724}$ of the susceptible and resistant *E. coli* isolates under the treatment of CTX, CAZ and LVX at their respective concentrations show a common behavior (Fig. 3). The obtained ROC curves (Figure S6) then yielded their excellent SERS-AST results in Table 2: the AUCs for the three drugs are all unity and the agreement rates between the results of SERS-AST and VITEK 2 are all 100%.

**Table 2:** SERS-AST results of gram-negative bacteria. ***N***_**S**_ and ***N***_**R**_ stand for numbers of susceptible and resistant cases, respectively

Bacterium	Antibiotic	${N}_{\text{S}}$		${N}_{\text{R}}$	${r}_{\text{O}\text{P}}^{*}$	AUC	${N}_{\text{D}\text{A}}$	${R}_{\text{A}}$ (%)
*E. coli*	CTX	14	6		0.65	1	0	100
	CAZ	14	6		0.53	1	0	100
	LVX	14	6		0.72	1	0	100
*E. cloacae*	CTX	12	8		0.68	0.96	1	95
	CAZ	12	8		0.69	0.98	1	95
* K. pneumoniae*	CTX	12	9		0.18	0.98	1	95
	CAZ	12	9		0.13	0.91	3	86
	LVX	16	5		0.68	0.96	1	95
	IPM	15	3		0.37	1	0	100
* A. baumannii*	CAZ	10	10		0.56	1	0	100
	LVX	10	10		0.52	1	0	100
	IPM	10	10		0.49	1	0	100

${\varvec{r}}_{\mathbf{O}\mathbf{P}}^{\varvec{*}}$ is the optimal cut-off SERS-AST signal ratio. ${\varvec{r}}_{\mathbf{O}\mathbf{P}}^{\varvec{*}}$ is the optimal cut-off signal ratio obtained from ROC analysis. AUC is the area under the ROC curve. ${\varvec{N}}_{\mathbf{D}\mathbf{A}}$ is number of isolates that show a SERS-AST result disagreeing with its VITEK 2 result. ${\varvec{R}}_{\mathbf{A}}$ is agreement rate, ${\varvec{N}}_{\mathbf{D}\mathbf{A}}/\left({\varvec{N}}_{\mathbf{S}}+{\varvec{N}}_{\mathbf{R}}\right)$

Similar to the *E. coli* isolates, there was no LVX- and IPM-resistant *E. cloacae* isolate. The corresponding ${r}_{724}$ distributions of the *E. cloacae* and *K. pneumoniae* isolates under the treatment of CTX and CAZ also exhibit the common behavior (Fig. 3). All the ROC analysis results (ROC curves shown in Figure S6) yielded their respective SERS-AST results in Table 2. Most of the agreement rates are at least 95% except the *K. pneumoniae*-CAZ combination, indicating that the SERS-AST protocol was also successfully demonstrated on the other three Gram-negative isolates treated with the corresponding antibiotics. There is one major-error sample in each of the *E. cloacae*-CTX, *E. cloacae-*CAZ combination, *K. pneumoniae*-CTX and *K. pneumoniae-*LVX combinations, resulting in 95% agreement in these cases. Unlike the SERS-AST results of the Gram-positive bacterium-LVX combinations presented above, all the agreement rates of the Gram-negative bacterium-LVX combinations are high. Notably, there are three major-error samples in the *K. pneumoniae*-CAZ combination, resulting in 86% agreement. The corresponding ${r}_{724}$ values of the *K. pneumoniae* isolates resistant to CAZ are significantly lower than 1 and even overlap those of the isolates susceptible to the drug (Fig. 3G). The poor SERS-AST performance of the *K. pneumoniae*-CAZ combination is discussed later.

After the antibiotic treatment, the *A. baumannii* sample extracted from the wash procedure underwent an additional step of 30-minute water bath at 37℃ to boost its SERS signal. In contrast to the SERS spectra of the other Gram-negative species, the SERS spectrum of *A. baumannii* (Figure S5**D**) exhibits a prominent spectral peak at 654 cm^−1^, as its dominant secreted purine derivative is xanthine (Premasiri et al. 2016). A test was performed to monitor the evolution of the SERS signal at 654 cm^−1^ after the water bath (${S}_{654}$) with respect to that without the water bath (${S}_{654}^{0}$) at different temperatures. The results (Figure S7) show that ${S}_{654}/{S}_{654}^{0}$ at 25 °C is increased gradually and reaches ~ 2.5 after 90 min; it at 37 °C rises promptly to ~ 2.2 after 30-minute water bath and then grows gradually to ~ 2.5 times after 90 min; and finally, its behavior at 50 °C is similar except with larger variation. Consequently, the additional water bath at 37 °C for 30 min is a simple, quick method to increase the SERS signal of *A. baumannii*. One possible reason why *A. baumannii* secretes a smaller amount of purines and derivatives than the other Gram-negative bacteria is that the permeability through its outer membrane is about 1/100 of *E. coli* (Geisinger et al. 2019; Zgurskaya and Rybenkov 2020). The raised temperature is known to increase membrane fluidity (Mitchell and Silhavy 2019) and thus expectantly increases the permeability of the outer membrane of *A. baumannii*. Other environmental stresses (Raivio 2005) (e.g., changed pH) and added agents (e.g., cations (Vaara 1992) can be the alternative means to boost the permeability (Nikaido 2003).

### SERS-AST with Levofloxacin

Among the seven antibiotics selected in this study, we note that the resultant optimal cut-off signal ratio (${r}_{\text{O}\text{P}}^{*}$) with LVX (a quinolone antibiotic that averts bacterial DNA from unwinding and duplicating) is frequently higher than that with other antibiotics five (OXA, AMP, CTX, CAZ and IPM). For example, ${r}_{\text{O}\text{P}}^{*}$ of the *S. aureus* samples treated with LVX is significantly higher than that treated with OXA (0.83 vs. 0.36); similarly, ${r}_{\text{O}\text{P}}^{*}$ of the *K. pneumoniae* samples treated with LVX is also higher than that treated with CAZ (0.68 vs. 0.13). Such behavior is reflected by the fact that in both cases the signal ratio of the bacterial isolates susceptible to the treated antibiotic at its break-point concentration does not decrease significantly (Figs. 2B and 3H), indicating that the amount of live susceptible bacteria remains large after the antibiotic treatment and thus potentially increasing the false-resistant (major error) cases owing to inevitable SERS signal fluctuation and thus warranting further study. Does it mean that the incubation time of the antibiotic treatment is not long enough because the bacterial species under test responds slowly to the antibiotic treatment?

A test was performed to demonstrate how the signal ratio of the SERS biomarker of *E. faecalis* (${r}_{730}$) was varied with the treatment time of LVX. The result (Figure S8) shows that ${r}_{730}$ with the 2-hr treatment, ${r}_{730}\left(2 \text{h}\text{r}\right)$, decreases gradually with the increase in the antibiotic concentration (${D}_{\text{L}\text{V}\text{X}}$) and drops to its base level for ${D}_{\text{L}\text{V}\text{X}}=$ 4 mg/l; ${r}_{730}\left(3 \text{h}\text{r}\right)$ stays at ~ 0.75 for ${D}_{\text{L}\text{V}\text{X}}$ = 0.5 and 1 mg/l and drops promptly to ~ 0.1 for ${D}_{\text{L}\text{V}\text{X}}$ = 2 mg/l; ${r}_{730}\left(4 \text{h}\text{r}\right)$ stays at ~ 0.5 for ${D}_{\text{L}\text{V}\text{X}}$ = 0.5, 1 and 2 mg/l and decreases gradually to the base level for ${D}_{\text{L}\text{V}\text{X}}$ = 8 mg/l. That is, ${r}_{730}\left(2 \text{h}\text{r}\right)$, ${r}_{730}\left(3 \text{h}\text{r}\right)$ and ${r}_{730}\left(4 \text{h}\text{r}\right)$ of *E. faecalis* treated with LVX at the break-point concentration (${D}_{\text{B}\text{P}}$ = 2 mg/l) are 0.86, 0.57 and 0.28, respectively. Accordingly, the 3 or 4-hour incubation time is more appropriate for the SERS-AST on the *E. faecalis*-LVX combination. Given the fluctuations of the signal ratio, the best ${r}_{\text{O}\text{P}}^{*}$ value would be located at ~ 0.5 or below to mitigate both very major error and major error cases simultaneously—i.e., if ${r}_{\text{O}\text{P}}^{*}$ is too high, the fluctuated SERS signal of a resistant isolate could falsely be regarded as a susceptible one (very major error); if ${r}_{\text{O}\text{P}}^{*}$ is too low, the fluctuated SERS signal of a susceptible isolate could falsely be regarded as a resistant one (major error). According to Figs. 2 and 3, the signal-ratio distributions of the resistant isolates more often spread more extensive than those of the susceptible isolates, thus yielding ultimate ${r}_{\text{O}\text{P}}^{*}$ values mostly below 0.5.

LVX is a quinolone antibiotic that binds to gyrase- or topoisomerase IV-DNA cleavage complex to form permanent chromosomal breaks (Mitscher 2005). The non-reparable breaks can lead to cell death. Khodursky, Zechierich and Cozzarelli concluded from the analysis of *E. coli* that gyrase is the primary target for quinolones while topoisomerase IV is the secondary one (Khodursky et al. 1995). With mutated strains, they discovered that the cell response of the primary target to the antibiotic is prompt—the cell viability decreases to < 10% within 10 min, while that of the secondary target is much slower—it takes ~ 3 h. for the cell viability to drop to 50%. That is, the bacterial response to quinolones depends on its target. Previous studies also showed that the target contribution to the quinolone action is varied on species-by-species and drug-by-drug basis (Aldred et al. 2014), and probably also on sample-by-sample basis owing to larger variation in clinical samples. In the *E. faecalis*-LVX combination (Figure S8), the antibiotic treatment time has to be at least 3 h. to show significant cell death probably because the bacterial response to the antibiotic may be slow. On the other hand, the bacterial response to the antibiotic is expectantly prompt in the *S. epidermidis*- and *A. baumannii*-LVX combinations, because their ${r}_{\text{O}\text{P}}^{*}$ values are 0.43 and 0.52, respectively. Therefore, the antibiotic treatment time of 3 or 4 h. for LVX and likely other quinolones is needed.

## Discussion

Based on the demonstration of the SERS-AST protocol—including (1) sample preparation, (2) antibiotic treatment, (3) SERS measurement, and (4) ROC analysis—for blood-culture isolates developed in our previous work on the *S. aureus*-OXA and *E. coli*-CTX combinations (Han et al. 2020), we have extended its application to the other 18 bacterium-antibiotic combinations, covering the additional 3 Gram-positive species (*S. epidermidis*, *E. faecalis* and *E. faecium*) and 3 Gram-negative species (*E. cloacae*, *K. pneumoniae* and *A. baumannii*) and the extra five antibiotics (VAN, AMP, CAZ, LVX and IPM). The resultant mean agreement rates for Gram-positive and Gram-negative species are 96% and 97%, respectively, indicating that the SERS-AST protocol can be successfully applied to these bacterium-antibiotic combinations and is expected to be equally applicable to other untested combinations. Two slight modifications are however needed to incorporate the differences in bacterial species and in antibiotics. First, additional 30-min incubation of the *A. baumannii* sample in water at 37 °C was needed to boost its secreted xanthine to account for the lower permeation efficiency of its outer membrane. Second, extended antibiotic treatment of LVX to compensate its slower mechanism of action effect is recommended to engender a prominent change of the SERS biomarker signal with respective to the antibiotic concentration and thus can produce more consistent AST outcomes. In sum, these results suggest that this simple general SERS-AST protocol can be implemented easily in hospital to shortening the waiting time to obtain highly accurate antibiotic-resistance reports. Nevertheless, there are still several issues that are worthy of further investigation.

In SERS-AST, bacterial response to antibiotics is determined by responsive changes in SERS signal, while conventional ASTs are mainly based on changes in bacterial number, which can be determined by OD_600_ readings. In this study, most of the tested cases show that the behavior of the optical density ratios—the ratio between the bacterial number after overnight incubation with and without mixing with a designated antibiotic—is consistent with that of the SERS signal ratios, as exemplified in the *S. epidermidis*-OXA combination (Figure S9**A**). We found that approximately 30% of the AST results measured by OD_600_ values were inconsistent with those determined by SERS-AST or VITEK 2. Most of the disagreement cases were Gram-negative bacteria treated with *β*-lactam antibiotics, such as the ones in the *E. cloacae*-CTX combination. It has been shown that the initial response of *E. cloacae* to the treatment of a *ꞵ*-lactam antibiotic is cell elongation, instead of decreased number of live cells (Cushnie et al. 2016). Therefore, the OD_600_ value of the treated culture may not change within 2 h of treatment for the *E. cloacae*-CTX combination (Figure S9**B**). Such morphological change is postulated to be a repair process for survival (Mitchell and Silhavy 2019). In contrast, SERS measures the change in the amounts of secreted purines and their derivatives in response to antibiotic treatment. Such measurement is less affected by changes in cell morphology and could more accurately reflect drug sensitivity in a short period of time.

LVX tests accounted for half of the disagreement cases, mostly with Gram-positive bacteria (Table 1). Among the seven antibiotics tested, LVX is the only one not acting on cell wall synthesis. It is a quinolone antibiotic that inhibits gyrase and topoisomerase IV leading to impaired DNA replication, repair, and recombination (Drlica et al. 2008). It has been shown that gyrase is the primary target of quinolones in Gram-negative bacteria, while topoisomerase IV is the main target of quinolones in Gram-positive bacteria (Drlica et al. 2008). In DNA replication, inhibition occurs within minutes when the antibiotic acts on gyrase (Snyder and Drlica 1979) but takes place later if it targets topoisomerase IV (Khodursky et al. 1995). However, bacterial responses to quinolones have been shown to vary on a species-by-species and drug-by-drug basis (Aldred et al. 2014). The widely spreading ${r}_{730}$ distributions of the *E. faecium*-LVX combination (Fig. 2H) suggest that the responses of *E. faecium* to quinolones could even vary on a strain-by-strain basis. It is likely that a longer time is required for LVX to obtain consistent responses from the Gram-positive bacteria. Based on the result shown in Figure S8, we recommend that the antibiotic treatment time be extended to at least 3 h for all the samples treated with LVX in future studies.

* Klebsiella pneumoniae* tested with CAZ, CTX, or LVX conferred the most disagreement cases (36%) (Table 2). *K. pneumoniae* is known to produce a pronounced polysaccharide capsule covering the entire bacterial surface resulting in a mucoid phenotype (Struve and Krogfelt 2003) with reduced ability to secrete purines and their derivatives, thus yielding weak SERS signals. *K. pneumoniae* is also known to produce the CTX M *β*-lactamase, which is an extended-spectrum *β*-lactamase (ESBL) and can degrade third-generation cephalosporin antibiotics at different rates (Canton et al. 2012). In addition, compared to CTX, which is the preferred target of CTX M *β*-lactamase, CAZ is relatively resistant to that enzyme and may require more than 2 h. to be degraded. Therefore, some CAZ-resistant bacteria may be determined by laboratory testing as susceptible, which is inconsistent with clinical manifestations. We also note a previous study (Roosendaal et al. 1987) showed that the bacterial count of *K. pneumoniae* treated with CAZ in broth decreases more slowly than the one treated with another more effective antibiotic (such as ciprofloxacin in that work). The Advanced Expert System (AES) of VITEK 2 can identify bacteria with ESBL by special software and modify the primary laboratory results accordingly (Spanu et al. 2006). In this study, there were five AES-revised *K. pneumoniae*-CAZ results. Three of them that were determined as susceptible by SERS-AST after 2-hour antibiotic incubation were interpreted by AES as resistant. This result thus raises a question whether a similar system like AES in VITEK 2 is also needed for SERS-AST.

Given the successful outcomes of SERS-AST in expediting the AST process without overnight incubation, there are still several issues for further investigation. First, can the treatment time of antibiotics be shortened? Adopting new culture media and condition may increase growth rate, adding cations and adjusting pH condition may boost the release of purines and derivative, and new SERS substrates and new optical detection schemes may help distinguish smaller difference between the SERS biomarker signals of susceptible and resistant strains. These three research directions are expected to yield a shorter incubation time in the SERS-AST protocol. Second, *P. aeruginosa* is another pathogen that shows high occurrence rate (5.3%) and critical antibiotic-resistant priority (by WHO) but was not studied here. The permeation efficiency of its cell envelope is as poor as *A. baumannii* and moreover the strong fluorescence emitted by its intracellular pigment (pyoverdine) (Cunrath et al. 2016) can easily overwhelm the weak SERS signal. Similarly, about 10% bacteremia arises from fungus infection (such as *Candida albicans*). A new sample preparation procedure would be needed to tackle the different biological characteristics and behaviors of these two pathogens. Third, polyclonal infection (PI) did occur in a few clinical cases in this study and thus raises the questions about the interpretation of the obtained SERS-AST result. Can the SERS spectrum be used to identify the responsible species in the PI case? Can the SERS spectrum after the antibiotic treatment be used to determine the respective resistograms of the co-present pathogens? More development of the current SERS-AST protocol to tackle the PI cases is needed. Fourth, in the whole SERS-AST protocol, 2 h are needed for sample washing and concentration adjustment in the pretreatment of blood-culture samples and the post-treatment of incubated samples with antibiotics, and about an hour is needed to adjust bacterial concentration for SERS measurement. The automation of these steps would certainly shorten the total operation time of the protocol.

## Conclusions

The emergency of new pathogens and antimicrobial resistance worldwide has stimulated the development of new antibiotics and fast diagnostic methods for precise and timely antimicrobial administration. Based on our previous successful demonstrations of the antimicrobial susceptibility testing with surface-enhanced Raman scattering (SERS-AST protocol) on blood-culture *S. aureus* and *E. coli* isolates treated with oxacillin and cefotaxime, respectively, we in this work have further extended the application of this protocol to 18 other bacterium-antibiotic combinations, including three new Gram-positive species (*S. epidermidis*, *E. faecalis* and *E. faecium*), three new Gram-negative species (*E. cloacae*, *K. pneumoniae* and *A. baumannii*), and five new antibiotics (levofloxacin, vancomycin, ampicillin, ceftazidime and imipenem). From the total 401 tests of blood-culture isolates, overall 96% agreement was achieved between the results of the SERS-AST protocol, obtained within five hours, and the VITEK 2 based on overnight incubation. This outcome proves that the SERS-AST protocol is a simple general AST method that can provide opportune and accurate AST information. This study also shows the developed and proposed strategies to accommodate unique biological characteristics and activities of bacterial species (low permeability of *A. baumannii* envelope) and distinctive mechanisms of action of antibiotics (elongated action of levofloxacin). These strategies can be easily incorporated to the protocol and are not expected to significantly influence the AST performance of all other bacterium-antibiotic combinations. The success of this study implies that this new AST paradigm based on prompt bacterial metabolism could potentially revolutionize the AST process.

## Supplementary Information

Below is the link to the electronic supplementary material.

Supplementary material 1 (DOCX 887.0 kb)

## Data Availability

The datasets used and analyzed in the current study are available from the corresponding author upon reasonable request.

## Declarations

### Source of bacterial samples

The bacterial species tested in the current study were obtained directly from blood culture of bacteremia patients.
