# Supplementary material for: SERS-based rapid susceptibility testing of commonly administered antibiotics on clinically important bacteria species directly from blood culture of bacteremia patients
Source: World J Microbiol Biotechnol. 2023 Aug 17;39(10):282. doi: 10.1007/s11274-023-03717-x (PMC10435613; doi:10.1007/s11274-023-03717-x)
Supplement: Supplementary file 1 — Supplementary material 1 (DOCX 887.0 kb) [file 11274_2023_3717_MOESM1_ESM.docx]

Supplementary Information

# SERS-Based Rapid Susceptibility Testing of Commonly Administered Antibiotics on Clinically Important Bacteria Species Directly from Blood Culture of Bacteremia Patients

Yin-Yi Han^1,2,3*^, Jann-Tay Wang^4,5^, Wei-Chih Cheng^6^, Ko-Lun Chen^6^, Yi Chi^6^, Lee-Jene Teng^7^, Juen-Kai Wang^6,8*^ & Yuh-Lin Wang^6*^

^1^Department of Anesthesiology, National Taiwan University Hospital, 7 Zhongshan S. Road, Taipei, 100225, Taiwan

^2^Department of Surgery, National Taiwan University Hospital, 7 Zhongshan S. Road, Taipei, 100225, Taiwan

^3^Department of Traumatology, National Taiwan University Hospital, 7 Zhongshan S. Road, Taipei, 100225, Taiwan

^4^Division of Infectious Diseases, Department of Internal Medicine, National Taiwan University Hospital, 7 Zhongshan S. Road, Taipei, 100225, Taiwan

^5^Taiwan National Institute of Infectious Diseases and Vaccinology, National Health Research Institutes, 35 Keyan Road, Zhunan, Miaoli, 35053, Taiwan

^6^Institute of Atomic and Molecular Sciences, Academia Sinica, 1 Roosevelt Road Sec. 4, Taipei, 10617, Taiwan

^7^Department of Clinical Laboratory Sciences and Medical Biotechnology, National Taiwan University, 1, Roosevelt Road Sec. 4, Taipei, 10048, Taiwan

^8^Center for Condensed Matter Sciences, National Taiwan University, 1 Roosevelt Road Sec. 4, Taipei, 106319, Taiwan

***Corresponding Authors**: YYH, yyhan@ntuh.gov.tw; JKW, jkwang@ntu.edu.tw; YLW, ylwang@pub.iams.sinica.edu.tw

# Table S1 Drug Concentrations of bacterium-antibiotic combinations for SERS-AST. Seven antibiotics—vancomycin (VAN), oxacillin (OXA), ampicillin (AMP), levofloxacin (LVX), cefotaxime (CTX), ceftazidime (CAZ) and imipenem (IMP)—and eight bacterium species—*S. aureus*, *S. epidermidis*, *E. faecalis*, *E. faecium*, *E. coli*, *E. cloacae*, *K. pneumoniae* and *A. baumannii* were selected. Four drug concentrations of each drug listed, in unit of mg/l, are in ascending order and varied by a factor of two between adjacent ones. The numbers in paratheses are the break-point concentrations chosen for ROC analysis.

|  | OXA | | AMP | LVX | VAN | CTX | CAZ | IPM |
| --- | --- | --- | --- | --- | --- | --- | --- | --- |
| *S. aureus* | 0.5~4 (2) | | – | 0.5~4 (1) | 1~8 (2) | – | – | – |
| *S. epidermidis* | 0.0625~0.5 (0.25) | | – | 0.5~4 (1) | 2~16 (4) | – | – | – |
| *E. faecalis* | – | | 4~32 (8) | 1~8 (2) | 2~16 (4) | – | – | – |
| *E. faecium* | – | | 4~32 (8) | 1~8 (2) | 2~16 (4) | – | – | – |
| *E. coli* | – | | – | 1~8 (2) |  | 0.5~4 (1) | 2~16 (4) | 0.5~4 (1) |
| *E. cloacae* | – | | – | 1~8 (2) |  | 0.5~4 (1) | 2~16 (4) | 0.5~4 (1) |
| *K. pneumoniae* | – | | – | 1~8 (2) |  | 0.5~4 (1) | 2~16 (4) | 0.5~4 (1) |
| *A. baumannii* | | – | – | 1~8 (2) | – | – | 4~32 (8) | 1~8 (2) |

**
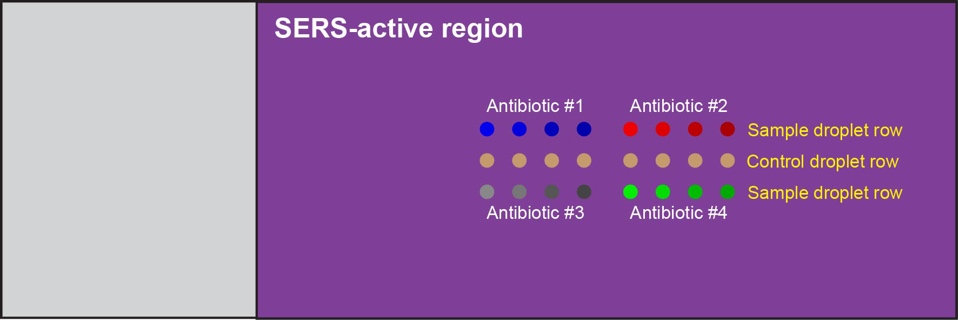
**

**Figure S1** **Layout of sample droplets on SERS substrate.** The sample droplets are laid on the SERS-active region of AgNP/AAO slide. The droplet diameter is ~1.5 mm. The separations between adjacent sample droplets are 2.5 mm. The brown circles are droplets of bacterial samples without antibiotic (control droplets); The four blue circles are the bacterial samples treated with Antibiotic #1 of four different concentrations; the four red circles are the ones treated with Antibiotic #2; the four gray circles are the ones treated with Antibiotic #3; the four green circles are the ones treated with Antibiotic #4. The darkness level of the colored circles reflects the antibiotic concentration: a darker level represents a higher concentration.


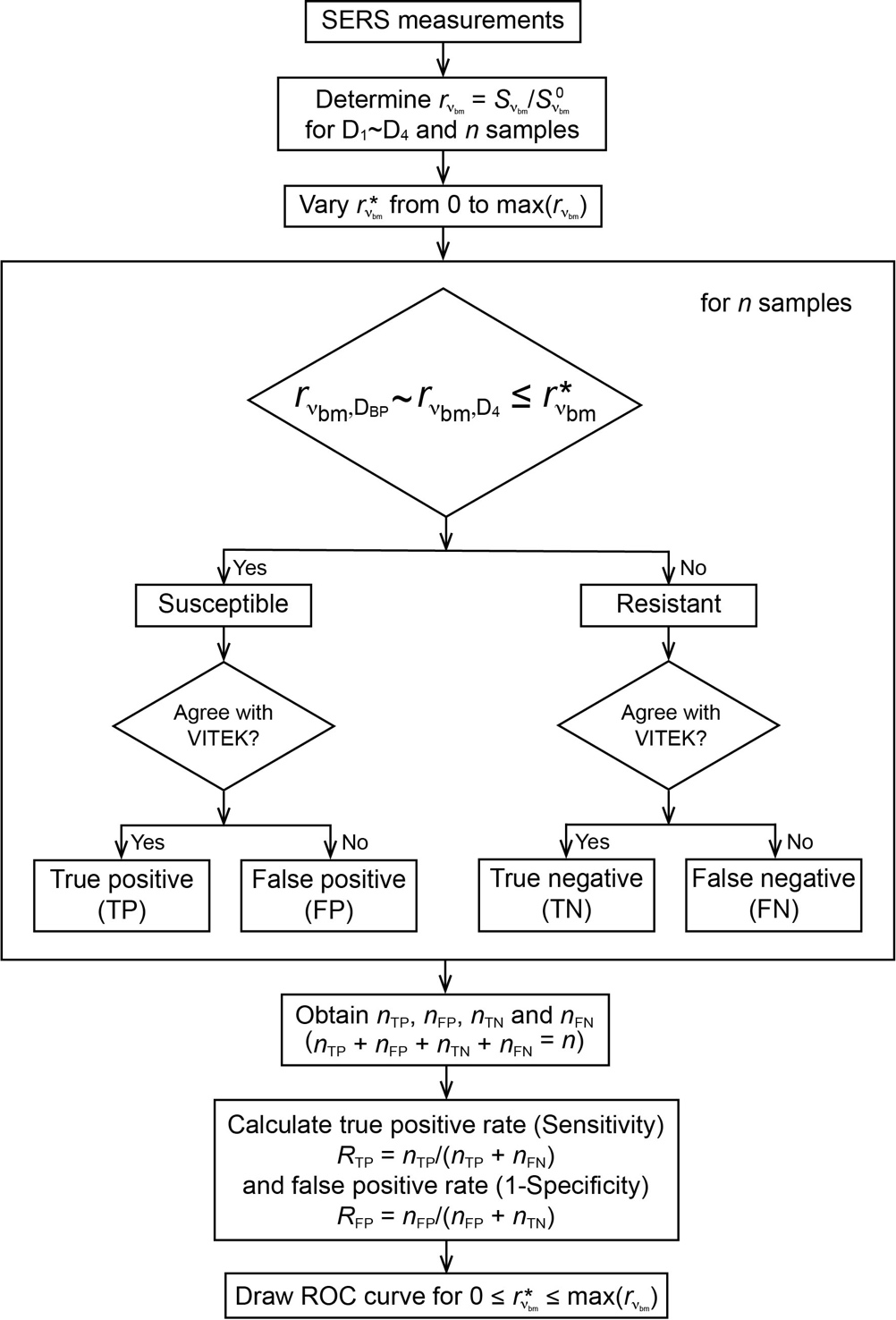


**Figure S2** **Flow chart of receiver operating characteristic (ROC) analysis.** $S_{\nu_{\mathrm{bm}}}^{D}$and $S_{\nu_{\mathrm{bm}}}^{0}$are the SERS signals at the Raman shift of the biomarker peak,$\nu_{\mathrm{bm}}$, with and without antibiotic treatment, respectively. $\nu_{\mathrm{bm}}$ is 730 cm^-1^ for Gram-positive bacteria of *S. aureus*, *S. epidermidis*, *E. faecalis* and *E. faecium*, is 724 cm^-1^ for Gram-negative bacteria of *E. coli*, *E. cloacae* and *K. pneumoniae*, and is 654 cm^-1^ for *A. baumannii*. $r_{\nu_{\mathrm{bm}}}$is the ratio between $S_{\nu_{\mathrm{bm}}}^{D}$and $S_{\nu_{\mathrm{bm}}}^{0}$. D_1_ indicates the lowest drug concentration, D_2_ indicates the second lowest drug concentration, and so on. $r_{\nu_{\mathrm{bm}}}^{*}$ is the cutoff signal ratio. $r_{\nu_{\mathrm{bm}},D_{j}}$is the signal ratio at $\nu_{\mathrm{bm}}$ of the bacterial sample treated with the antibiotic of concentration $D_{j}$. $n_{\mathrm{TP}}$,$n_{\mathrm{FP}}$, $n_{\mathrm{TN}}$ and $n_{\mathrm{FN}}$are the numbers of true positive, false positive, true negative and false negative cases, respectively. *M* is the total number of samples. $\max\left( r_{\nu_{\mathrm{bm}}} \right)$ is the maximal measured $r_{\nu_{\mathrm{bm}}}$ obtained from the *M* samples.

**
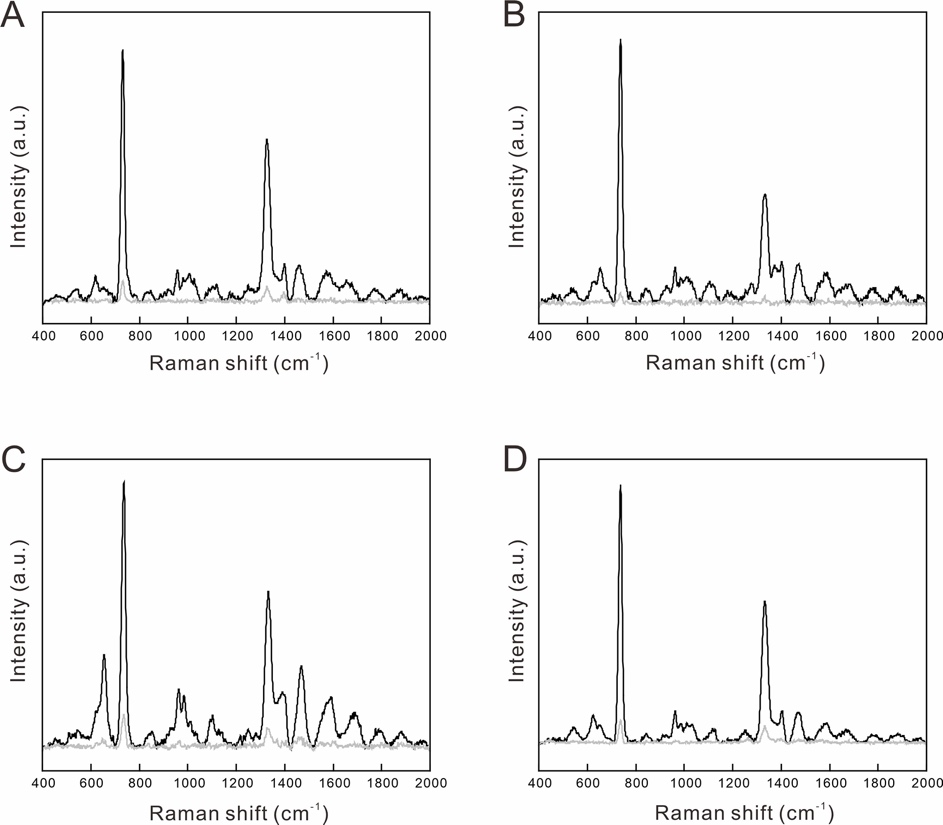
**

**Figure S3** **SERS spectra of blood-cultured Gram-positive bacteria.** **A** is for *S. aureus*, **B** is for *S. epidermidis*, **C** is for *E. faecalis* and **D** is for *E. faecium*. The gray curves underneath show their corresponding standard deviation.

**
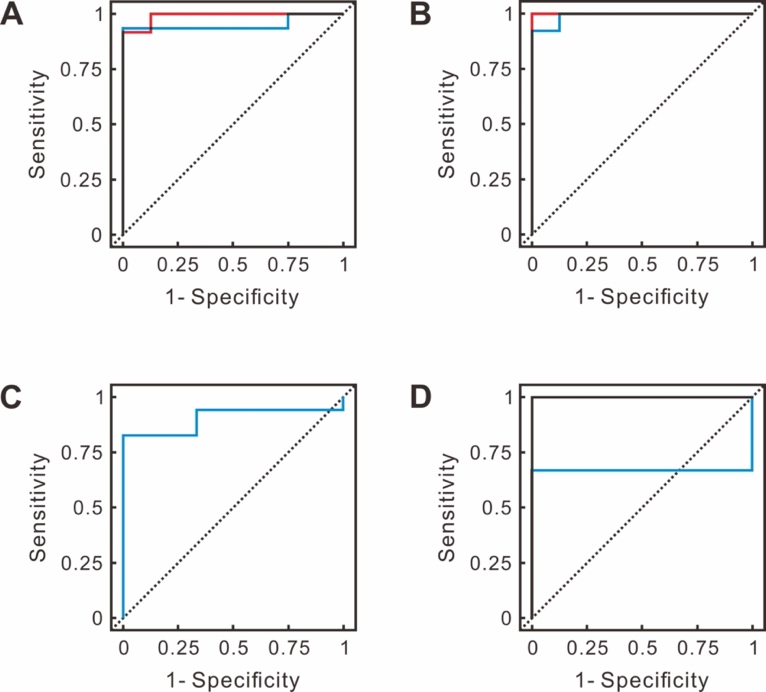
**

**Figure S4** **ROC curves of SERS-AST results of Gram-positive bacteria. A** is for *S. aureus*, **B** is for *S. epidermidis*, **C** is for *E. faecalis* and **D** is for *E. faecium*. The red, blue, green and orange curves are for OXA, LVX, AMP and VAN, respectively, while the black segments are overlap.


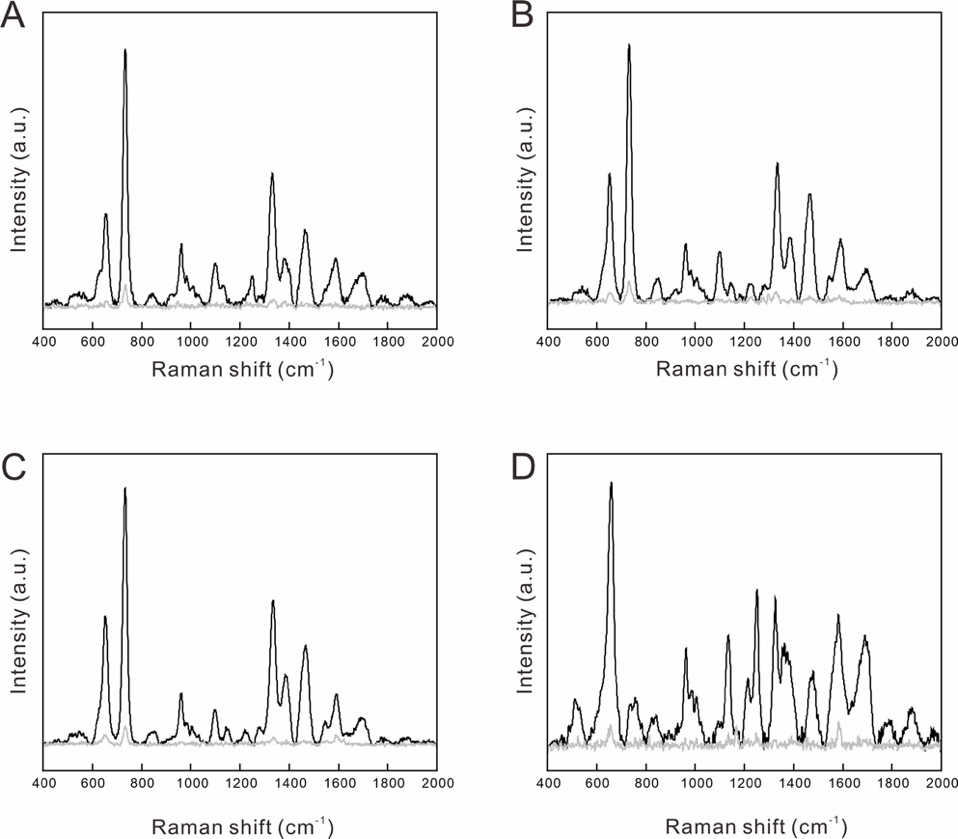


**Figure S5** **SERS spectra of blood-cultured Gram-negative bacteria.** **A** is for *E. coli*, **B** is for *E. cloacae*, **C** is for *K. pneumoniae* and **D** is for *A. baumannii*. The black curves are the spectra of susceptible species. The gray curves underneath show their corresponding standard deviation.

**
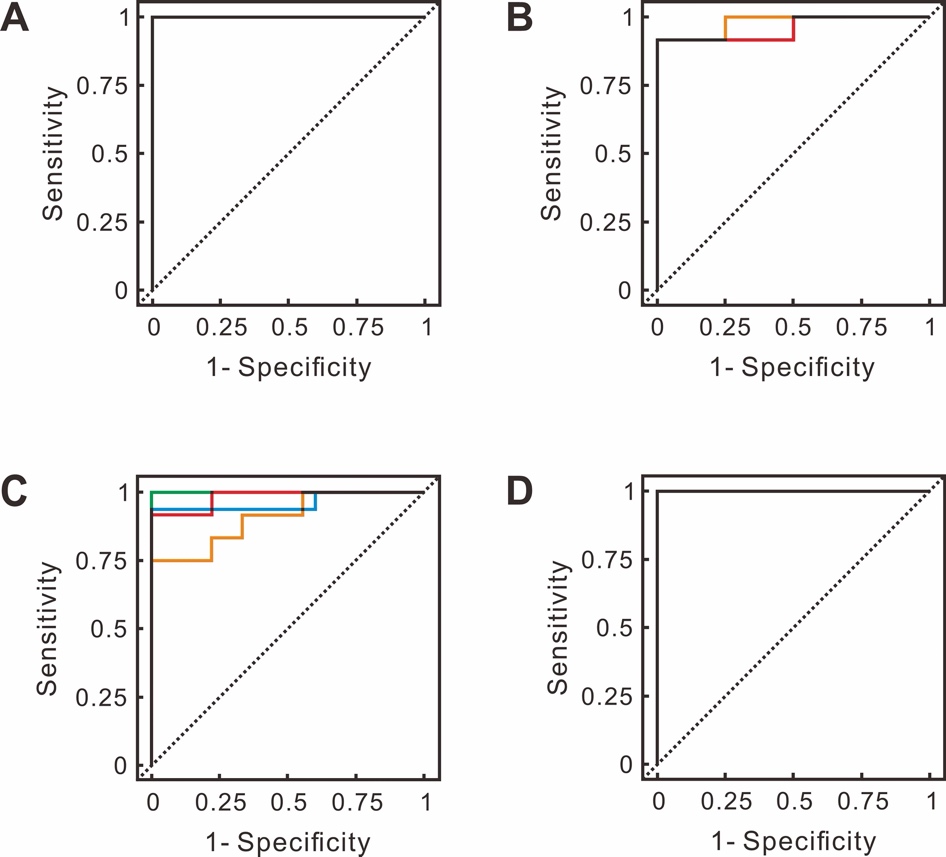
**

**Figure S6 ROC curves of SERS-AST results of Gram-negative bacteria. A** is for *E. coli*, **B** is for *E. cloacae*, **C** is for *K. pneumoniae* and **D** is for *A. baumannii*. The red, blue, green and orange curves are for CTX LVX, IPM and CAZ, respectively, while the black segments show overlap.


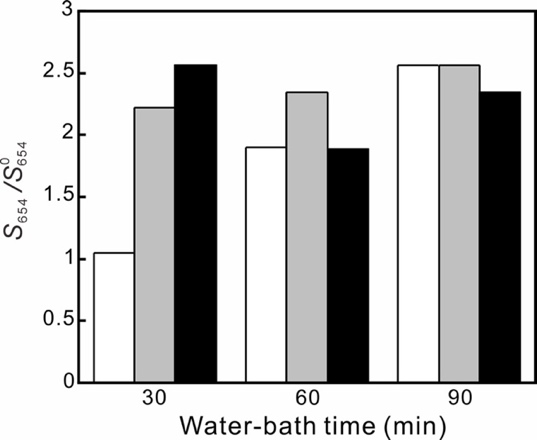


**Figure S7** **SERS biomarker signal of *A. baumannii* with extra water bath.** $S_{654}$ is the SERS signal of *A. baumannii* at 654 cm^-1^ obtained with different incubation conditions and $S_{654}^{0}$ is that obtained without extra incubation. White, gray and black columns represent the incubation at 25, 37 and 50°C, respectively.


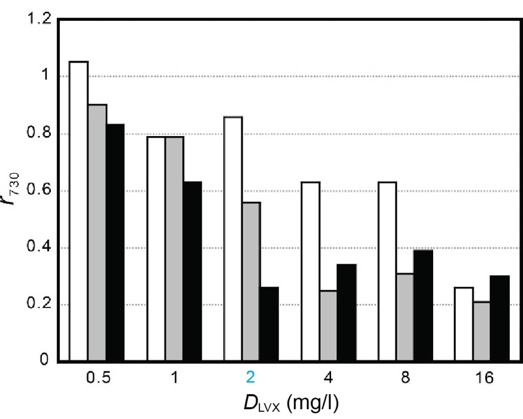


**Figure S8** **Treatment time test of** ***E. faecalis*-LVX.** $r_{730}$ is the signal ratio calculated by dividing the signal intensity at 730 cm^-1^ of the antibiotic-treated sample by that of the non-treated control. $D_{\mathrm{LVX}}$ is LVX concentration. The break-point drug concentration of the *E. faecalis*-LVX combination is 2 mg (Table S1). White, gray, and black columns represent the data of different LVX treatment time: 2 hours, 3 hours, and 4 hours, respectively.


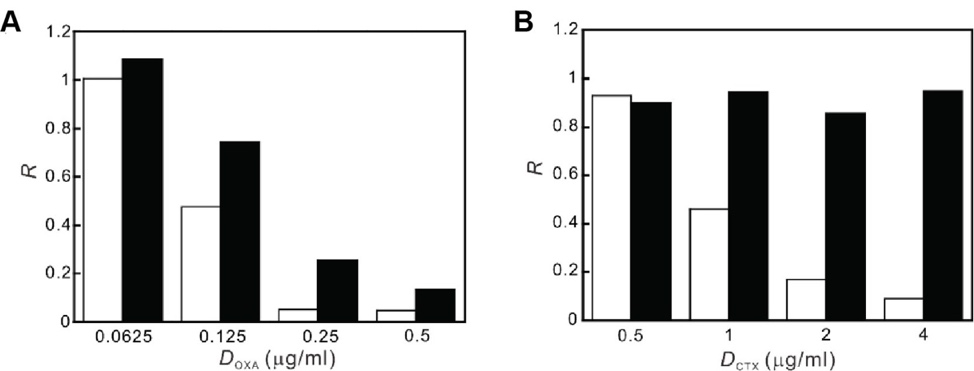


**Figure S9** **SERS-AST vs. optical density. A** is SERS-AST signal and OD_600_ ratios of *S. epidermidis* obtained with four OXA concentrations; **B** is SERS-AST signal and OD_600_ ratios of *E. cloacae* obtained with four CTX concentrations. White columns denote the ratio of the SERS signal with the antibiotic treatment to that without the treatment, while black columns denote the corresponding ratio of the OD_600_ value.
